# Autistic-Like Traits in Adult Patients with Mood Disorders and Schizophrenia

**DOI:** 10.1371/journal.pone.0122711

**Published:** 2015-04-02

**Authors:** Junko Matsuo, Yoko Kamio, Hidetoshi Takahashi, Miho Ota, Toshiya Teraishi, Hiroaki Hori, Anna Nagashima, Reiko Takei, Teruhiko Higuchi, Nobutaka Motohashi, Hiroshi Kunugi

**Affiliations:** 1 Department of Mental Disorder Research, National Institute of Neuroscience, National Center of Neurology and Psychiatry, Kodaira, Tokyo, Japan; 2 Department of Child and Adolescent Mental Health, National Institute of Mental Health, National Center of Neurology and Psychiatry, Kodaira, Tokyo, Japan; 3 Department of Adult Mental Health, National Institute of Mental Health, National Center of Neurology and Psychiatry, Kodaira, Tokyo, Japan; 4 National Center of Neurology and Psychiatry, Kodaira, Tokyo, Japan; 5 Department of Neuropsychiatry, Interdisciplinary Graduate School of Medicine and Engineering, University of Yamanashi, Chuo, Yamanashi, Japan; Chiba University Center for Forensic Mental Health, JAPAN

## Abstract

Autism spectrum disorder often co-occurs with other psychiatric disorders. Although a high prevalence of autistic-like traits/symptoms has been identified in the pediatric psychiatric population of normal intelligence, there are no reports from adult psychiatric population. This study examined whether there is a greater prevalence of autistic-like traits/symptoms in patients with adult-onset psychiatric disorders such as major depressive disorder (MDD), bipolar disorder, or schizophrenia, and whether such an association is independent of symptom severity. The subjects were 290 adults of normal intelligence between 25 and 59 years of age (MDD, n=125; bipolar disorder, n=56; schizophrenia, n=44; healthy controls, n=65). Autistic-like traits/symptoms were measured using the Social Responsiveness Scale for Adults. Symptom severity was measured using the Positive and Negative Symptoms Scale, the Hamilton Depression Rating Scale, and/or the Young Mania Rating Scale. Almost half of the clinical subjects, except those with remitted MDD, exhibited autistic-like traits/symptoms at levels typical for sub-threshold or threshold autism spectrum disorder. Furthermore, the proportion of psychiatric patients that demonstrated high autistic-like traits/symptoms was significantly greater than that of healthy controls, and not different between that of remitted or unremitted subjects with bipolar disorder or schizophrenia. On the other hand, remitted subjects with MDD did not differ from healthy controls with regard to the prevalence or degree of high autistic-like traits/symptoms. A substantial proportion of adults with bipolar disorder and schizophrenia showed high autistic-like traits/symptoms independent of symptom severity, suggesting a shared pathophysiology among autism spectrum disorder and these psychiatric disorders. Conversely, autistic-like traits among subjects with MDD were associated with the depressive symptom severity. These findings suggest the importance of evaluating autistic-like traits/symptoms underlying adult-onset psychiatric disorders for the best-suited treatment. Further studies with a prospective design and larger samples are needed.

## Introduction

Autism spectrum disorder (ASD) is an early-onset, life-long developmental disorder characterized by persistent deficits in social reciprocity and social communication, as well as restricted, repetitive patterns of behaviors, interests, or activities [1]. The prevalence of ASD in children and adolescents is estimated to be in the range of 0.6–0.7% [autistic disorder (0.2%), pervasive developmental disorder-not otherwise specified (PDD-NOS) (0.3%), and Asperger’s disorder (0.06%)], and has continually increased over the last 15–20 years [2].

It has been recognized that youth with ASD often have co-occurring psychiatric disorders [3–5]. Such co-occurrence may negatively impact social and academic performance, even for those who have an intelligence quotient (IQ) within the normal range [6]. There have been many reports on the incidence of psychiatric comorbidity with ASD in clinical pediatric cases, although the findings vary widely [4]. There has been only one population-based study, which was conducted in the UK, for 112 children with ASD aged 10 to 14 years [3]. This study revealed that approximately 70% of children with ASD suffered from at least one comorbid Diagnostic Statistical Manual (DSM) axis-I psychiatric disorder such as social anxiety disorder, attention-deficit/hyperactivity disorder (ADHD), and oppositional defiant disorder (30% each). Approximately 10% of children with ASD had experienced a period of depression or irritability, despite the fact that major depressive disorder (MDD) or dysthymic disorder was identified in less than 1% of children with ASD. Psychotic disorders such as schizophrenia (SZ) or bipolar disorder (BPD) were not observed in this sample.

On the other hand, frequent co-occurrence of ASD among psychiatric patients has been identified in several studies of clinical youth aged 7–17 years that had been diagnosed with mood or anxiety disorders [7,8]. Pine et al. [8] observed that 57% of youth patients with BPD, 38% with MDD, and 25% with anxiety disorder exceeded the clinical cut-off for quantitative ASD scales. These findings suggest that autistic-like traits/symptoms (ALTs) as measured by quantitative ASD scales are closely associated with mood and anxiety disorders in pediatric patients, although the etiological relevance is unclear.

The psychosocial outcomes of adults who were diagnosed with ASD in childhood tend to range from poor or very poor in young adulthood (<25 years) [9], to even worse in later adulthood, although the severity of ASD decreases throughout development [10]. Reduced psychosocial quality of life (QOL) in high-functioning adults with ASD was found to be associated with concurrent behavioral and psychiatric problems [11]. Although psychiatric comorbidity, as well as core symptoms, seems to hamper the social adaptation of adults with ASD, there is only one study on the incidence of psychiatric comorbidity in adults with ASD. Hofvander et al. [12] examined patients who visited their clinic or hospital, which specialize in childhood-onset neuropsychiatric disorders, and reported that all patients of normal intelligence that were diagnosed with ASD during childhood (n = 122) had at least one life-time comorbid DSM axis-I psychiatric disorder: the most prevalent was mood disorders (53%), followed by anxiety disorders (50%), ADHD (43%), obsessive-compulsive disorder (24%), chronic tic disorders (20%), substance-related disorders (16%), and psychotic disorders (12%).

Similarly, studies of the non-clinical adult population also provided evidence of the association between ALTs and psychiatric conditions such as depression and anxiety [13, 14]. This is consistent with findings in the non-clinical child population: a sub-clinical level of ALTs was associated with emotional, behavioral, and cognitive problems [15, 16].

To our knowledge, the prevalence of ALTs among the adult psychiatric population outside ASD clinics has not been reported. In Japan, an increasing number of adults with ALTs visit general psychiatric clinics with a diverse range of chief complaints, seeking accurate diagnosis and/or treatment for concurrent psychiatric symptoms [17]. However, unlike children diagnosed with ASD, clinical manifestations in adulthood are often complex: core symptoms tend to become less apparent [9], or adults with ASD may use compensatory strategies to mask their deficits. For these reasons, clinical or subclinical ASD symptoms are likely to be overlooked in general psychiatric settings, which can lead to inappropriate treatment [5,18].

The aim of this study was to examine whether higher levels of ALTs are associated with adult-onset psychiatric disorders (i.e., MDD, BPD, and SZ), and whether such an association is independent of symptom severity. We hypothesized that higher ALT levels would be frequently observed among psychiatric adult patients, similar to rates observed among children/adolescents. Regarding the association between ALT degree and symptom severity of non-ASD psychiatric disorders, no specific predictions were made.

## Methods

### Subjects

Subjects were 290 adults (men, 48%) aged 25 to 59 years. They consisted of 44 patients with SZ (men, 46%), 125 with MDD (56%), 56 with BPD (46%), and 65 healthy controls (HC) (28%) (Table 1). They were recruited through notices posted in the National Center of Neurology and Psychiatry (NCNP) Hospital, website announcements, or advertisements in a local free paper. All subjects other than HC were outpatients who attended either the NCNP hospital or local hospitals/clinics. All subjects were interviewed by a trained psychiatrist using the Japanese version of the Mini-International Neuropsychiatric Interview [19,20], and diagnoses were confirmed based on the DSM of Mental Disorders, 4^th^ ed., text rev. (DSM-IV-TR) [21]. Schizophrenic symptoms were corroborated by administering the Positive and Negative Symptoms Scale (PANSS) to all subjects with SZ [22]. Depression severity was assessed using the 17-item version of the Hamilton Depression Rating Scale (HDRS-17) for all subjects with MDD and BPD [23]. Manic symptoms were assessed by the Young Mania Rating Scale (YMRS) [24] for all subjects with BPD. Based on the definition of remission determined by the International Society for Bipolar Disorders Task Force [25], subjects with BPD whose YMRS total scores were 8 or over were considered to be suffering from a significant manic state and excluded from this study. Subjects with MDD and BPD were divided into remitted and unremitted subgroups based on the total score of the HDRS-17 (≤7: remitted; >7: unremitted). Likewise, subjects with SZ were divided into two subgroups based on their PANSS scores according to the criteria proposed by Andreasen et al. [26]; to meet remission criteria, a subject’s scores for all of the following items must remain under 4 for at least 6 months, and he/she must not have been hospitalized during that period: delusion, conceptual disorganization, hallucinatory behavior, blunt affect, passive apathetic social withdrawal, lack of spontaneity and flow of conversation, mannerisms and posturing, unusual thought content. Those already given a clinical diagnosis of ASD were excluded. However, we did not conduct a thorough and comprehensive evaluation of ASD diagnostics for the present study. In addition, those who had intellectual disability were excluded. Full-scale IQs were assessed using the Wechsler Adult Intelligence Scale-Third edition (WAIS-III) [27] for 66 subjects with MDD (53%), 33 with BPD (59%), 36 with SZ (82%), and 58 with HC (89%); the remaining subjects were clinically judged as functioning within normal range.

**Table 1:** Demographics, clinical data and SRS-A total raw scores of MDD, BPD, SZ and HC.

	MDD (n = 125)	BPD (n = 56)	SZ (n = 44)	HC (n = 65)	Analysis (χ^2^/ANOVA/t)	Significant pair-wise comparison
***Demographics***						
Men (%)	70 (56%)	26 (46%)	20 (46%)	18 (28%)	χ^2^(3) = 14, *p* = 0.003	MDD, SZ, BPD>HC
					χ^2^(2) = 2.2, n.s.(among patient groups)	
Age (years)						
M ± SD	41.5 ±9.2	40.4 ±7.8	36.9 ±7.5	42.2 ±8.2	F(3, 286) = 3.9, *p* = 0.01	HC, MDD>SZ
Education (years)						
M ± SD	15.3 ±2.0	15.5 ±3.4	13.8 ±2.4	15.1 ±2.5	F(3, 286) = 4.6, *p* = 0.004	BPD, MDD>SZ
***Clinical variables***						
Full-scale IQ^a^						
M ± SD	109.1 ±11.7	105.5 ±14.6	96.2 ±12.1	111.4 ±13.4	F(3,189) = 11.5, *p<*0.001	HC,MDD,BPD>SZ
Range	70–133	70–138	75–116	87–136		
Medication (mg/day)						
AD M ± SD^b^	155.9 ±230.5	161.3 ±227.3	54.5 ±164.9	-	F(2, 198) = 3.8, *p* = 0.024	BPD,MDD>SZ
AP M ± SD^c^	55.8 ±130.2	89.3 ±169.3	467.3 ±530.8	-	F(2, 198) = 35.8, *p<*0.001	SZ>BPD,MDD
Remitted N (%)	46 (37%)	20 (36%)	14 (32%)		χ^2^(2) = 0.35, n.s.	
HDRS-17 M ± SD	11.1 ±7.5	11.4 ±7.9	-	-	t(179) = 0.3, n.s.	
YMRS M ± SD	-	1.3 ±1.8	-	-	-	
PANSS M ± SD						
positive	-	-	14.1 ±4.4	-	-	
negative	-	-	16.1 ±5.3	-	-	
general psycho-pathol.	-	-	31.6 ±8.6	-	-	
total	-	-	61.8 ±15.3	-	-	
**SRS-A total score**						
Mean (SD)	48.7 (25.3)	55.4 (25.8)	59.6 (25.0)	32.5 (19.1)	F(2, 286) = 14.0, *p<*0.001	SZ,BPD,MDD>HC**
Median ± Q^d^	43.0 (15.5)	53.0 (18.5)	60.0 (21)	29.0 (11.5)	χ^2^(3) = 41.1, *p<*0.001	SZ,BPD>HC*** SZ>MDD**>HC***

^a^Full-scale IQs were measured for 66 with MDD (53%), 33 with BPD (59%), 36 with SZ (82%) and 58 HC (89%).

^b^imipramine equivalent dose of anti-depressant.

^c^chlorpromazine equivalent dose of anti-psychotics.

^d^Kruskal Wallis test was used for the effect of diagnosis on the SRS-A total raw scores; Mann-Whitney U test for between-group differences.

MDD: major depressive disorder. BPD: bipolar disorder. SZ: schizophrenia. HC: healthy control.

HDRS: Hamilton Depression Rating Score. YMRS: Young Mania Rating Scale. PANSS: Positive and Negative Symptoms Scale.

M: Mean. SD: Standard Deviation. Q: Quartile deviation. n.s. p>0.05

This study was approved by the National Center of Neurology and Psychiatry Ethics Committee (23–185). Written informed consent was obtained from all participants prior to their inclusion in the study.

### Autistic-like traits assessment

The Social Responsiveness Scale for Adults (SRS-A) [28] was distributed to either a family member or a close friend of the subject. The SRS-A is a quantitative measure of ALTs for 19 to 59-year-old adults. Similar to the original SRS [29], which was developed for 4 to 18-year-olds, the SRS-A is to be completed by a family member or a person who knows the subject well enough to provide an accurate account of his/her behaviors during the preceding 6 months. The SRS-A contains 65 Likert-scaled (0–3) items (0: never, 1: sometimes, 2: often, 3: almost always) that are divided into the following 5 subscales: social awareness, social cognition, social communication, social motivation, and autistic mannerisms. The scores are reported to be distributed widely and continuously in the general population from normal to the extreme end of the autistic spectrum [30–34]. According to the original manual [29], T-scores of 59T or less are defined as within the normal range, while those of 60T through 75T are defined as within the mild-to-moderate range, typical for high-functioning ASD such as Asperger’s disorder and PDD-NOS, and possibly causing substantial interference with everyday social interactions. T-scores of 76T or higher are defined as within the severe range and are typical for autistic disorder and more severe PDD-NOS, indicating severe interference with everyday social interactions. In this study using the Japanese version [34], we classified two subgroups based on SRS-A T-score [33]: one with 59T or less (low ALT: L-ALT) and another with 60T or higher (high ALT: H-ALT). A T-score of 60T (which is equivalent to raw score ≥53) derived from the Japanese standardization sample corresponds to approximately the 87th percentile in our HC sample, indicating its representativeness. These T-scores were based on combined data from both sexes, because SRS-A raw scores in the standardization sample revealed no statistically significant sex differences among adults 25–59 years old [34]. This was reconfirmed in the current study.

### Statistical analysis

Statistical analyses were performed using SPSS version 22.0 (SPSS Japan, Tokyo). The proportion of categorical variables was compared using the chi-squared test. Demographic information (age, and years of education) and clinical information (IQ, and medication) were compared across diagnoses by one-way analysis of variance (ANOVA) and between remitted and unremitted subgroups within each diagnosis by the Student’s *t*-test. Effects of these variables on SRS-A score were examined by multiple regression analysis. Since SRS-A data were skewed, non-parametric analyses were used for the subsequent analyses. The SRS-A raw scores were compared among the 7 subgroups (HC and remitted and unremitted subgroups of MDD, BPD, and SZ) using a Kruskal-Wallis test; between-group differences were examined by a pairwise multiple comparison test using rank sums proposed by Dunn [35]. To examine the association between SRS-A raw scores and the symptom severity of each psychiatric disorder, Spearman’s correlation coefficients were used between the HDRS-17 and SRS-A raw scores in the MDD and BPD groups, and between the PANSS and SRS-A raw scores for patients with SZ. Statistical significance was set at a two-tailed *p*<0.05.

## Results

### Demographic and clinical characteristics

**Fig 1:**
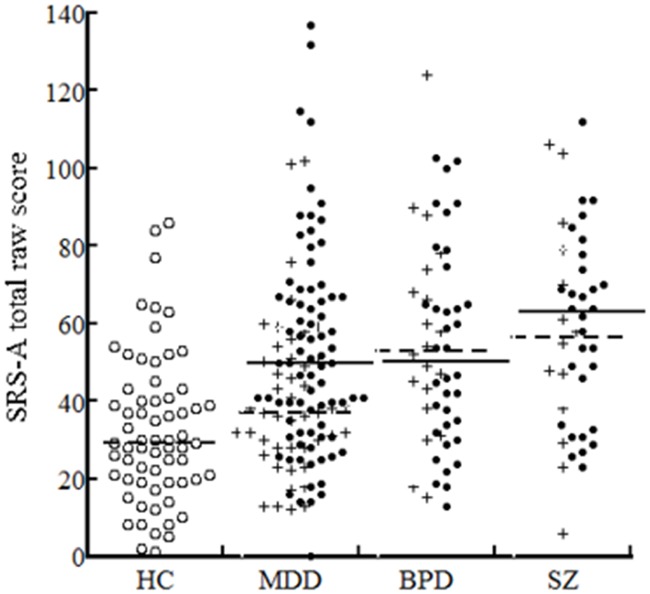
Dot plots of the SRS-A total raw scores for the 7 subgroups. Bar indicates median score: solid line for unremitted subjects and broken line for remitted subjects. Solid dot indicates unremitted subjects and cross indicates remitted subjects. HC: healthy control. MDD: major depressive disorder. BPD: bipolar disorder. SZ: schizophrenia.

The demographic and clinical characteristics of the subjects are shown in Table 1. The male to female ratio was significantly higher in the MDD, SZ, and BPD groups than in the HC, but did not differ across the three clinical groups. Age, education, IQ, and medication differed significantly between the SZ group and the remaining three groups (MDD, BPD, and HC); however, multiple regression analysis in which SRS-A raw score was a dependent variable and these demographic and clinical variables were explanatory variables yielded no significant effects (*p*>0.05). The ratio of remitted versus unremitted subjects was not significantly different among the MDD, BPD, and SZ groups. Between remitted and unremitted subgroups within each diagnosis, there were no significant differences in demographics or clinical variables (age, years of education, IQ, age of onset, duration of illness, history of being hospitalized, hospitalized months, and recurrent episode) (S1 Table). Since the effect of sex on SRS-A raw scores was not significant in either diagnosis (S2 Table), data derived from both sexes were combined in the subsequent analyses. The distribution of SRS-A total raw scores is shown by diagnostic group in Fig 1.

### Comparison of the degree of ALTs among the 7 subgroups

**Fig 2:**
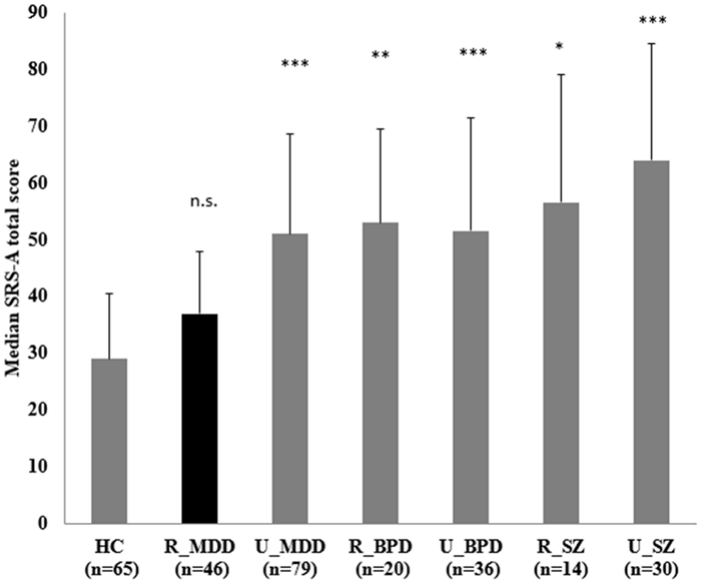
Comparison of the degree of ALTs among the 7 subgroups. SRS-A total scores were compared among the 7 subgroups (i.e., HC and remitted/unremitted MDD, BPD and SZ) using Kruskal Wallis test; Dunn's test was used for further pair-wise multiple comparison. Results of pair-wise comparison between each clinical subgroup and HC are shown with error bar showing quartile deviation. HC: healthy control. MDD: major depressive disorder. BPD: bipolar disorder. SZ: schizophrenia. R: remitted. U: unremitted. n.s. *p*>0.05, * *p*<0.05, ** *p*<0.01, *** *p*<0.001 (all against HC)

All the clinical subgroups, whether subjects were remitted or unremitted, except for the remitted MDD subgroup, had significantly higher total and social communication and autistic mannerisms subscale scores on the SRS-A compared to the HC group (Fig 2; S3 Table). The remitted MDD subgroup scored significantly lower overall and on the social cognition and social communication subscales relative to the unremitted SZ group, and scored lower on the social motivation subscale than did the unremitted MDD subgroup (as highlighted in S3 Table).

### Comparison of the proportion of H-ALTs among the 7 subgroups

The number and percentage of H-ALTs in each subgroup are shown in S4 Table. The proportion of H-ALTs in the remitted MDD group (22%) was significantly lower compared to that in the unremitted MDD group (46%) (*χ* ^2^ = 7.1, two-tailed *p* = 0.012), but did not differ significantly from that of the HC group (14%). The proportion of H-ALTs in the BPD (50%), SZ (61%) and unremitted MDD groups was significantly higher than that of the HC and remitted MDD groups, but did not significantly differ from each other. The BPD and SZ subjects did not differ regarding the proportion of H-ALTs between remitted and unremitted subgroups.

### Associations between ALTs and symptom severity of psychiatric disorders

**Fig 3:**
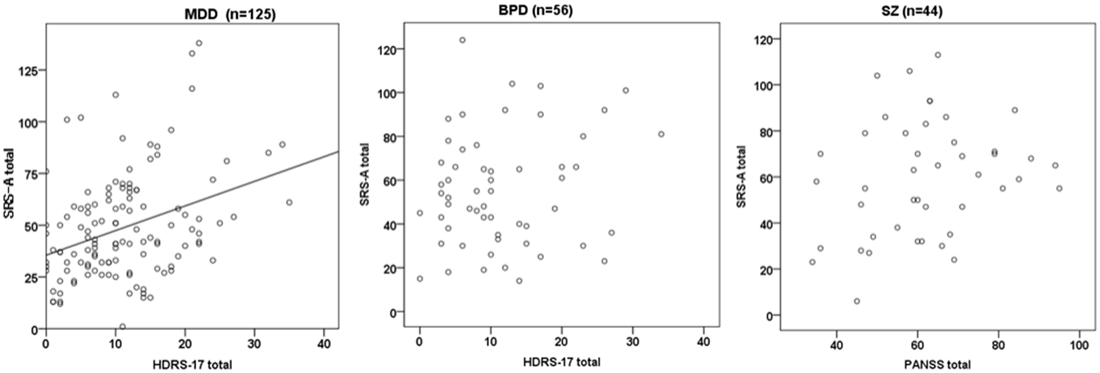
Scatter plots of SRS-A total raw score and symptom severity. Significant correlation was found between the SRS-A and HDRS-17 total in the MDD group (*r* = 0.32, *p*<0.001), but not in the BPD group (*r* = 0.16, n.s.). No correlation was found between the SRS-A and PANSS total in the SZ group (*r* = 0.25, n.s.).

In the MDD group, including remitted and unremitted subjects, SRS-A raw scores were moderately correlated with HDRS-17 total score (*r* = 0.32, *p*<0.001). On the contrary, no significant correlations between the SRS-A and HDRS-17 total scores were found for the BPD group, or between the SRS-A and PANSS total scores for the SZ group. Scatter plots are shown for the MDD, BPD, and SZ groups (Fig 3).

## Discussion

To our knowledge, this is the first study to examine the distribution of ALTs in the adult psychiatric population diagnosed with MDD, BPD, or SZ, and the possible association between ALTs and psychiatric symptomatology.

We found that adult subjects diagnosed with MDD, BPD, or SZ exhibited a higher degree of ALTs compared to HCs. Almost half of the clinical subjects, except those with remitted MDD, fell into the mild-to-severe range for ALTs, which is typical for sub-threshold or threshold ASD. This finding suggests an aggregation of ALTs in individuals with adult-onset psychiatric disorders, and together with the findings about concurrent psychiatric disorders in clinical youths [7,8,16,29], implies a possible pathophysiological overlap between ASD and other psychiatric disorders. Furthermore, our findings may be in accordance with evidence from a recent genetic study examining risk loci demonstrating shared effects on ASD, ADHD, BPD, MDD, and SZ [36], with a stronger polygenic overlap between ASD and SZ and between ASD and BPD rather than between ASD and MDD. Similarly, neurocognitive and phenomenological commonalities between ASD and SZ [18, 37, 38] and ASD and BPD [39] have been noted in the literature. However, behavioral commonality between ASD and adult-onset psychiatric disorders observed in the present study does not necessarily imply etiological commonality. Interpretative caution must be taken as future research emerges.

In our study, the degree of ALTs in BPD and SZ subjects was not associated with symptom severity. Further, the proportion of subjects with high ALTs did not significantly differ between remitted and unremitted subjects in the BPD and SZ groups. Thus, ALTs as measured by the SRS-A appear independent of psychiatric symptoms in this population, and we consequently conclude that ALTs in our psychiatric population (except for MDD subjects) remain at a high level regardless of the symptom severity of the psychiatric disorder.

On the other hand, ALTs in subjects with MDD were associated with the depressive symptom severity in our study; in other words, although subjects with severe depressive symptoms tended to exhibit high ALTs, subjects with less severe depressive symptoms did not differ from healthy controls with regard to the proportion or degree of high ALTs. It is difficult to explain this finding because we did not examine intra-individual differences. The reason may be complicated because ASD and depression have several common symptoms such as social withdrawal and obsessionality. However, one possible interpretation would be that the ALTs shown by some depressive patients may decrease when depression is remitted, but resurface when depressive symptoms worsen, which would lead to misdiagnosis confounding ALTs with depressive symptoms [40]. This possibility is also mentioned in the SRS manual [28]; however, there is no evidence for this at present.

There are several limitations to this study. First, a significantly lower male to female ratio in the HC relative to the patient groups may have exaggerated the true differences between these groups. However, we confirmed that the effect of sex on SRS-A raw scores was not significant for either patient group or the HC group. Second, we did not conduct a thorough and comprehensive evaluation of ASD during our exclusion procedure. Therefore, we cannot deny the possibility that some individuals undiagnosed with ASD in the present study could have met the ASD diagnostic criteria. This is because SRS-A scores for some patients exceeded the cutoff [34]. Although we found an overlap between high ALTs and adult-onset psychiatric disorders, irrespective of the diagnostic status for ASD in the present study, replication studies should be conducted assessing adult non-ASD psychiatric patients via more thorough diagnostic procedures. Third, the sample size was relatively small. Fourth, this was a cross-sectional study; however, we did confirm that remitted and unremitted subgroups in this study did not significantly differ in terms of demographic or clinical variables. Nonetheless, our findings need to be re-examined by performing a longitudinal study. Fifth, the demographic variables of patients with SZ were not matched to those of other diagnostic patients, although it was a natural discrepancy. However, as these variables had no significant effect on SRS-A score, it is unlikely that this discrepancy affected our results.

To conclude, the presentation of ALTs at the sub-threshold or threshold level may be closely associated with BPD and SZ. High ALTs were observed irrespective of symptom severity in a subgroup of subjects with BPD and SZ, suggesting a shared pathophysiology among BPD, SZ, and ASD. Conversely, ALTs among subjects with MDD were associated with depressive symptom severity, which should be acknowledged when assessing ALTs among MDD patients. Whether ALTs observed among subjects with MDD are trait- or state-dependent should be examined in future studies using a prospective design with a larger sample. Our results stress the importance of clinicians’ attention to underlying sub-threshold or threshold ALTs in adult patients with MDD, BPD, and SZ. Untangling persistent ALTs from childhood to adulthood and adult-onset psychiatric disorders would be helpful in the choice of an appropriate treatment plan for such patients. In general psychiatric settings, the use of validated ASD symptom scales, such as the SRS-A, represents an easy and effective way to screen for sub-threshold or threshold ASD, determine accurate diagnosis, provide the best-suited treatment, and subsequently evaluate the effects of treatment.

## Supporting Information

S1 Table Demographics and clinical data of remitted and unremitted MDD, BPD, and SZ subjects. ^a^Hospitalized months were compared by Mann-Whitney U test. MDD: major depressive disorder. BPD: bipolar disorder. SZ: schizophrenia. PANSS: Positive and Negative Symptoms Scale. M: Mean. SD: Standard Deviation. n.s. p>0.05.
(XLSX)

S2 Table SRS-A total raw score by diagnosis and sex. ^a^Mann-Whitney U test was used to identify the effect of sex for each diagnosis. ^b^Chi-squared test was used to compare the proportion of L-ALT and H-ALT between diagnoses. M-W: Mann-Whitney U test. L-ALT: low autistic-like traits. H-ALT: high autistic-like traits. MDD: major depressive disorder. BPD: bipolar disorder. SZ: schizophrenia. HC: healthy controls. Q: quartile deviation. n.s. p>0.05.
(XLSX)

S3 Table Comparison of SRS-A total and subscale raw scores among the 7 subgroups. ^a^Kruskal Wallis test was used among the above 7 subgroups (i.e., HC and remitted/unremitted MDD, BPD, and SZ) to examine the effect of diagnosis on the SRS-A total and subscale scores. ^b^Pairwise multiple comparison test using rank sums proposed by Dunn OJ was used to further identify the between-group differences. HC: healthy control. MDD: major depressive disorder. BPD: bipolar disorder. SZ: schizophrenia. R: remitted. U: unremitted. Q: quartile deviation. * p<0.05, ** p<0.01, *** p<0.001.
(XLSX)

S4 Table Comparison of the proportion of H-ALTs among the 7 subgroups. ^a^Chi-squared test was used to compare the proportion of L-ALT and H-ALT between diagnoses. HC: healthy control. MDD: major depressive disorder. BPD: bipolar disorder. SZ: schizophrenia. R: remitted. U: unremitted. L-ALT: low autistic-like traits. H-ALT: high autistic-like traits. * p<0.05, ** p<0.01, *** p<0.001.
(XLSX)

## References

[1] American Psychiatric Association (2013) DSM-5th: Diagnostic and Statistical Manual of Mental Disorders, 5th ed Washington DC: American Psychiatric Press.

[2] Fombonne E. (2009) Epidemiology of pervasive developmental disorders. Pediatr Res 65(6): 591–8. 10.1203/PDR.0b013e31819e7203 19218885

[3] Simonoff E, Pickles A, Charman T, Chandler S, Loucus T, et al (2008) Psychiatric disorders in children with autism spectrum disorders: prevalence, comorbidity, and associated factors in a population-derived sample. J Am Acad Child Adolesc Psychiatry 47(8): 921–9. 10.1097/CHI.0b013e318179964f 18645422

[4] Skokauskas N, Gallagner L (2010) Psychosis, affective disorders and anxiety in autistic spectrum disorder: prevalence and nosological considerations. Psychopathology 43(1): 8–16. 10.1159/000255958 19893339

[5] Mazefsky CA, Oswald DP, Day TN, Eack SM, Minshew NJ, et al (2012) ASD, a psychiatric disorder, or both? Psychiatric diagnoses in adolescents with high-functioning ASD. J Clin Child Adolesc Psychol 41(4): 516–23. 10.1080/15374416.2012.686102 22642847 PMC3601822

[6] Kaat AJ, Gadow KD, Lecavalier L (2013) Psychiatric symptoms impairment in children with autism spectrum disorders. J Abnorm Child Psychol 41(6): 959–69. 10.1007/s10802-013-9739-7 23605958

[7] Towbin KE, Pradella A, Gorrindo T, Pine DS, Leibenluft E (2005) Autism spectrum traits in children with mood and anxiety disorders. J Child Adolesc Psychopharmacol 15(3): 452–64. 16092910 10.1089/cap.2005.15.452

[8] Pine DS, Guyer AE, Goldwin M, Towbin KA, Leibenluft E (2008) Autism spectrum disorder scale scores in pediatric mood and anxiety disorders. J Am Acad Child Adolesc Psychiatry 47(6): 652–61. 10.1097/CHI.0b013e31816bffa5 18434923 PMC2735817

[9] Howlin P, Moss P (2012) Adults with autism spectrum disorders. Can J Psychiatry 57: 275–83. 22546059 10.1177/070674371205700502

[10] Howlin P, Moss P, Savage S, Rutter M (2013) Social outcomes in mid- to later adulthood among individuals diagnosed with autism and average nonverbal IQ as children. J Am Acad Child Adolesc Psychiatry 52(6): 572–81. 10.1016/j.jaac.2013.02.017 23702446

[11] Kamio Y, Inada N, Koyama T (2013) A nationwide survey on quality of life and associated factors of adults with high-functioning autism spectrum disorders. Autism 17: 15–26. 10.1177/1362361312436848 22399449

[12] Hofvander B, Delorme R, Chaste P, Nydén A, Wentz E, et al (2009) Psychiatric and psychosocial problems in aduts with normal-intelligence autism spectrum disorders. BMC Psychiatry 9:35 10.1186/1471-244X-9-35 19515234 PMC2705351

[13] Kanne SM, Christ SE, Reiersen AM (2009) Psychiatric symptoms and psychosocial difficulties in young adults with autistic traits. Journal of Autism and Developmental Disorders 39:827–33. 10.1007/s10803-008-0688-x 19132522

[14] Kunihira Y, Senju A, Dairoku H, Wakabayashi A, Hasegawa T (2006) ‘Autistic’ traits in non-autistic Japanese populations: Relationships with personality traits and cognitive ability. Journal of Autism and Development Disorders 36(4): 553–66. 16602034 10.1007/s10803-006-0094-1

[15] Möricke E, Swinkels SH, Beuker KT, Buitelaar JK (2010) Predictive value of subclinical autistic traits at age 14–15 months for behavioural and cognitive problems at age 3–5 years. Eur Child Adolesc Psychiatry 19(8): 659–68. 10.1007/s00787-010-0103-y 20390313 PMC2910304

[16] Kamio Y, Moriwaki A, Takei R, Inada N, Inokuchi E, et al (2013) Psychiatric issues of children and adults with autism spectrum disorders who remain undiagnosed. Seishin Shinkeigaku Zasshi 115(6): 601–6 (in Japanese). 23944117

[17] Kamio Y, Inokuchi E (2009) Psychiatric practice’s role for individual with developmental disorders: current trend and future issues. Journal of Jpn Association of Psychiatric Hospitals 28: 14–20 (in Japanese).

[18] Cochran DM, Dvir Y, Frazier JA (2013) “Autism-plus” spectrum disorders: intersection with psychosis and the schizophrenia spectrum. Child Adolesc Psychiatric Clin N Am 22: 609–27. 10.1016/j.chc.2013.04.005 24012076

[19] Sheehan DV, Lecrubier Y, Sheehan KH, Amorim P, Janavs J, et al (1998) The Mini-International Neuropsychiatric Interview (M.I.N.I.): the development and validation of a structured diagnostic psychiatric interview for DSM-IV and ICD-10. J Clin Psychiatry 59 Suppl 20, 22–33: quiz 34–57. 9881538

[20] Otsubo T, Tanaka K, Koda R, Shinoda J, Sano N, et al (2005) Reliability and validity of Japanese version of the Mini-International Neuropsychiatric Interview. Psychiatry Clin Neurosci. 59(5): 517–26. 16194252 10.1111/j.1440-1819.2005.01408.x

[21] American Psychiatric Association (2000) DSM-IV-TR: Diagnostic and Statistical Manual of Mental Disorders, 4th ed, text rev. Washington DC: American Psychiatric Press.

[22] Kay SR, Fischizophreniabein A, Opler LA (1987) The positive and negative syndrome scale (PANSS) for schizophrenia. Schizophr Bull 13: 261 3616518 10.1093/schbul/13.2.261

[23] Hamilton M (1967) Development of a rating scale for primary depressive illness. Br J Soc Clin Psychol 6(4): 278–96. 6080235 10.1111/j.2044-8260.1967.tb00530.x

[24] Young RC, Biggs JT, Ziegler VE, Merger DA (1979) A rating scale for mania. Reliability, validity, sensitivity. Br J Psychiatry 133: 429–33. 10.1192/bjp.133.5.429 728692

[25] Tohen M, Frank E, Bowden CL, Colom F, Ghaemi SN, et al (2009) The International Society for Bipolar Disorders (ISBD) Task Force report on the nomenclature of course and outcome in bipolar disorders. Bipolar Disord. 11(5): 453–73. 10.1111/j.1399-5618.2009.00726.x 19624385

[26] Andreasen NC, Carpenter WT Jr, Kane JM, Lasser RA, Marder SR, et al (2005) Remission in schizophrenia: proposed criteria and rationale for consensus. Am J Psychiatry 162(3): 441–9. 15741458 10.1176/appi.ajp.162.3.441

[27] Wechsler D (1997) Wechsler Adult Intelligence Scale-III. San Antonio: The Psychological Corporation.

[28] Constantino JN, Gruber CP (2012) Social Responsiveness Scale, Second Edition (SRS-2). Los Angeles: Western Psychological Services.

[29] Constantino JN, Gruber CP (2005) Social Reponsiveness Scale (SRS). Los Angeles: Western Psychological Services.

[30] Constantino JN, Todd RD (2003) Autistic traits in the general population: a twin study. Arch Gen Psychiatry 60(5): 524–30. 12742874 10.1001/archpsyc.60.5.524

[31] Bölte S (2012) Brief Report: The Social Responsiveness Scale for Adults (SRS-A): Initial Results in a German Cohort. J. Autism Dev Disord 42: 1998–9. 10.1007/s10803-011-1424-5 22183423 PMC3425739

[32] Kamio Y, Inada N, Moriwaki A, Kuroda M, Koyama T, et al (2013) Quantitative autistic traits ascertained in a national survey of 22529 Japanese schoolchildren. Acta Psychiatr Scand 128(1): 45–53. 10.1111/acps.12034 23171198 PMC3604131

[33] Kamio Y, Takei R, Inada N, Kuroda M, Nakano I, et al (2013) Study on developing the multi-dimensional classification guidelines in each stage of life In: Uchiyama T, editor. Annual research report on developing the guidelines on the diagnosis from childhood to adulthood based on the longitudinal follow-up study of individuals with developmental disorders. Tokyo: National Center of Neurology and Psychiatry pp. 45–59 (in Japanese).

[34] Takei R, Matsuo J, Takahashi H, Uchiyama T, Kunugi H, et al (2014) Verification of the utility of the social responsiveness scale for adults in non-clinical and clinical adult populations in Japan. BMC Psychiatry 14:302. 25403232 10.1186/s12888-014-0302-z PMC4237729

[35] Dunn OJ (1964) Multiple comparisons using rank sums. Technometrics 6: 241–252.

[36] Cross-Disorder Group of the Psychiatric Genomics Consortium (2013) Identification of risk loci with shared effects on five major psychiatric disorders: a genome-wide analysis. Lancet 381 (9875): 1371–9. 10.1016/S0140-6736(12)62129-1 23453885 PMC3714010

[37] Chung YS, Barch D, Strube M (2014) A meta-analysis of mentalizing impairment in adults with schizophrenia and autism spectrum disorder. Schizophrenia Bulletin 40: 602–616. 10.1093/schbul/sbt048 23686020 PMC3984506

[38] de Lacy N, King BH (2013) Revisiting the relationship between autism and schizophrenia: toward an integrated neurobiology. Annu Rev Clin Psychol 9: 555–587. 10.1146/annurev-clinpsy-050212-185627 23537488

[39] Vannucchi G, Masi G, Toni C, Dell’Osso L, Erfurth A, et al (2014) Bipolar disorder in adults with Asperger’s Syndrome: a systematic review. J Affect Disord 168: 151–60. 10.1016/j.jad.2014.06.042 25046741

[40] Stewart ME, Barnard L, Pearson J, Hasan R, O’Brien G (2006) Presentation of depression in autism and Asperger syndrome: a review. Autism 10(1): 103–16. 16522713 10.1177/1362361306062013

